# The correlation of pericoronary adipose tissue with coronary artery disease and left ventricular function

**DOI:** 10.1186/s12872-022-02843-y

**Published:** 2022-09-06

**Authors:** Deshu You, Haiyang Yu, Zhiwei Wang, Xiaoyu Wei, Xiangxiang Wu, Changjie Pan

**Affiliations:** 1 Department of Radiology, The Affiliated Changzhou No. 2 People’s Hospital of Nanjing Medical University, Changzhou, 213003 Jiangsu China; 2 Department of Interventional and Vascular Surgery, The Affiliated Changzhou No. 2 People’s Hospital of Nanjing Medical University, Changzhou, 213003 China

**Keywords:** Pericoronary adipose tissue, Fat attenuation index, Left ventricular function, Gensini score, Coronary artery disease

## Abstract

**Objective:**

We sought to investigate the correlation of pericoronary adipose tissue with coronary artery disease and left ventricular (LV) function.

**Methods:**

Participants with clinically suspected coronary artery disease were enrolled. All participants underwent coronary computed tomography angiography (CCTA) and echocardiography followed by invasive coronary angiography (ICA) within 6 months. Pericoronary adipose tissue (PCAT) was extracted to analyze the correlation with the Gensini score and LV function parameters, including IVS, LVPW, LVEDD, LVESD, LVEDV, LVESV, FS, LVEF, LVM, and LVMI. The correlation between PCAT and the Gensini score was assessed using Spearman’s correlation analysis, and that between the PCAT volume or FAI and LV function parameters was determined using partial correlation analysis.

**Results:**

One hundred and fifty-nine participants (mean age, 64.55 ± 10.64 years; men, 65.4% [104/159]) were included in the final analysis. Risk factors for coronary artery disease, such as hypertension, diabetes, dyslipidemia, and a history of smoking or drinking, had no significant association with PCAT (*P* > 0.05), and there was also no correlation between PCAT and the Gensini score. However, the LAD-FAI was positively correlated with the IVS (r = 0.203, *P* = 0.013), LVPW (r = 0.218, *P* = 0.008), LVEDD (r = 0.317, *P* < 0.001), LVESD (r = 0.298, *P* < 0.001), LVEDV (r = 0.317, *P* < 0.001), LVESV (r = 0.301, *P* < 0.001), LVM (r = 0.371, *P* < 0.001), and LVMI (r = 0.304, *P* < 0.001). Also, the LCX-FAI was positively correlated with the LVEDD (r = 0.199, *P* = 0.015), LVESD (r = 0.190, *P* = 0.021), LVEDV (r = 0.203, *P* = 0.013), LVESV (r = 0.197, *P* = 0.016), LVM (r = 0.220, *P* = 0.007), and LVMI (r = 0.172, *P* = 0.036), and the RCA-FAI was positively correlated with the LVEDD (r = 0.258, *P* = 0.002), LVESD (r = 0.238, *P* = 0.004), LVEDV (r = 0.266, *P* = 0.001), LVESV (r = 0.249, *P* = 0.002), LVM (r = 0.237, *P* = 0.004), and LVMI (r = 0.218, *P* = 0.008), respectively. Finally, the total volume was positively correlated with FS (r = 0.167, *P* = 0.042).

**Conclusion:**

The FAI was positively correlated with the LV function but was not associated with the severity of coronary artery disease.

**Supplementary Information:**

The online version contains supplementary material available at 10.1186/s12872-022-02843-y.

## Key points

- A significant correlation exists between the fat attenuation index (FAI) and left ventricular function parameters.
- Our results suggest that cardiac mortality caused by an increased FAI may be due to changes in the left ventricular function.
- Pericoronary adipose tissue did not correlate with the body mass index or risk factors of coronary heart disease.

**Supplementary Information:**

The online version contains supplementary material available at 10.1186/s12872-022-02843-y.

## Introduction

Coronary artery disease (CAD) is a heart disease in which atherosclerotic plaque accumulates in the coronary artery, causing coronary lumen stenosis or occlusion and often resulting in myocardial ischemia or necrosis. Coronary atherosclerosis has been considered to be an inflammatory reaction [1]. For patients with CAD, predicting the risk of adverse coronary events is more important than coronary stenosis. The detection of pericoronary inflammation can facilitate risk stratification and risk prediction earlier in patients with coronary heart disease.

In recent years, the pericoronary fat attenuation index (FAI), which is measured using coronary CT angiography (CCTA) images, can be used to reflect pericoronary inflammation [2, 3]. Recent studies have shown that pericoronary adipose tissue (PCAT) participates in the process of coronary inflammation and is significantly related to the type of plaque and degree of stenosis [4–6]. One CRISP study showed that an FAI of >  − 70.1 HU was associated with a greater risk for cardiac mortality, which could improve the prediction of a heart disease risk [7].

Although PCAT was shown to have great clinical significance in the occurrence and development of CAD [8], there are several important questions that must be explored in this popular field. The Gensini score is a traditional index used to evaluate the severity of CAD [9]; however, it is unknown whether PCAT assessment can determine the severity of CAD. Second, some studies reported that CAD is a cause of left ventricular (LV) dysfunction, and LV dysfunction can increase the mortality of CAD, but the association between PCAT and LV function is still unclear [10, 11].

The aim of this study was to investigate the association of PCAT with CAD and LV function, which can help clinicians to evaluate the prognosis of patients with CAD.

## Methods

This retrospective study was approved by the ethics committee of Changzhou No. 2 People's Hospital affiliated with Nanjing Medical University, and the need to obtain informed consent was waived.

### Study patients

**Fig. 1:**
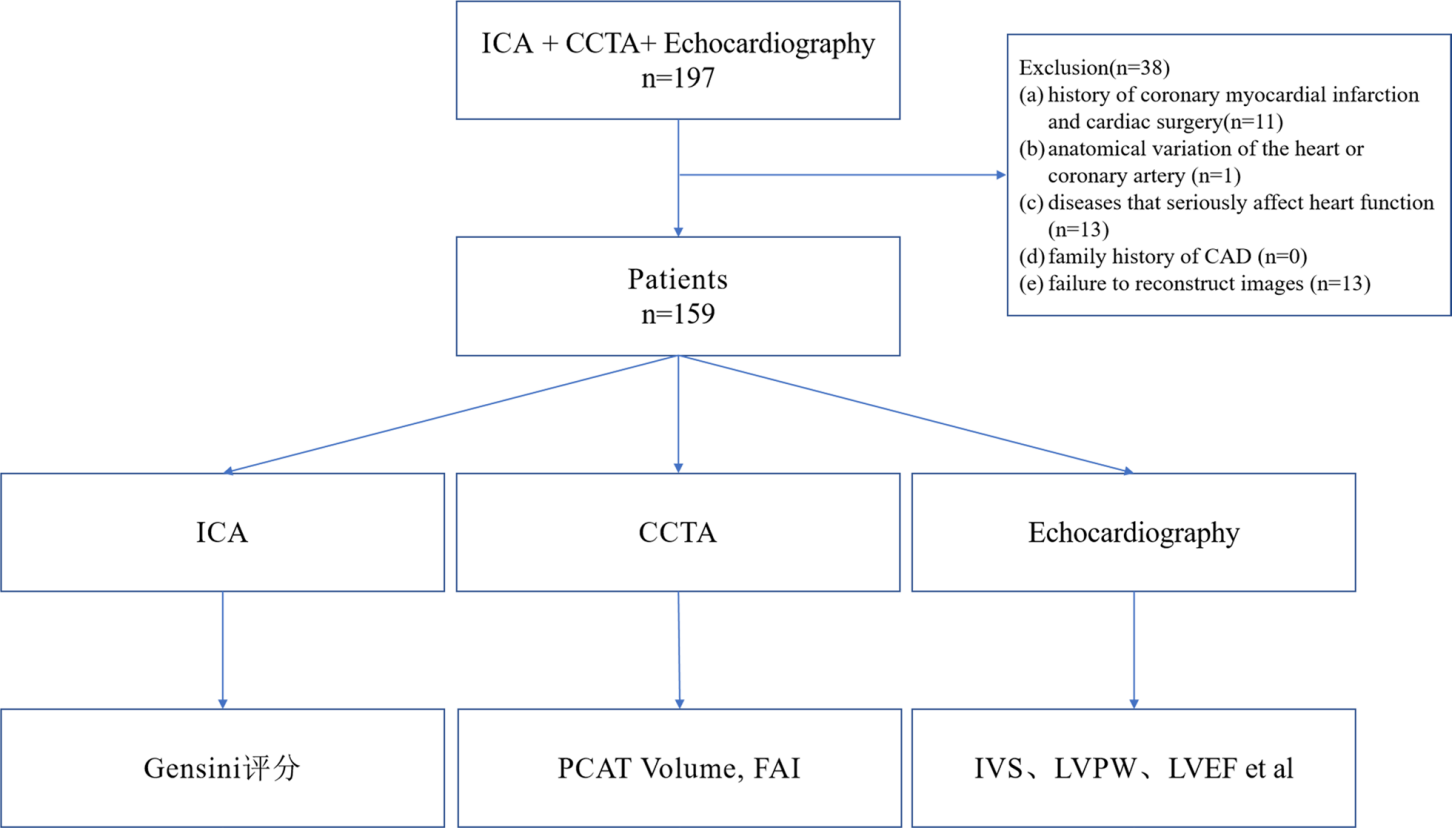
Flowchart of study inclusion and exclusion

The study subjects were all patients treated at Changzhou No. 2 People's Hospital affiliated with Nanjing Medical University from September 2019 to September 2021. We retrospectively included patients with clinically suspected CAD in our institution who underwent CCTA and echocardiography followed by invasive coronary angiography (ICA) within 6 months. Exclusion criteria included the following: (a) history of coronary myocardial infarction and cardiac surgery; (b) anatomical variation of the heart or coronary artery; (c) diseases that seriously affect heart function, such as heart space–occupying lesions, cardiomyopathy, or severe heart valve disease; (d) family history of CAD; and (e) failure to reconstruct images (Fig. 1).

### Clinical data

The clinical data of patients were obtained from the inpatient care system. Hypertension, diabetes, and dyslipidemia were diagnosed according to clinical indicators. A diagnosis of hypertension was defined by a systolic blood pressure ≥ 140 mmHg and/or diastolic blood pressure ≥ 90 mmHg or taking anti-hypertensive drugs. The diagnosis of diabetes was made based on the guideline for the prevention and treatment of type 2 diabetes and defined by a fasting blood glucose > 6.1 mmol/L and/or glycosylated hemoglobin > 6.5% or hypoglycemic drugs. Dyslipidemia was diagnosed according to the guidelines for the prevention and treatment of dyslipidemia in adults and defined as meeting ≥ 1 of the following requirements: (a) total cholesterol ≥ 5.18 mmol/L, (b) triglyceride ≥ 1.70 mmol/L, (c) high-density lipoprotein cholesterol < 1.04 mmol/L, (d) low-density lipoprotein cholesterol ≥ 3.37 mmol/L, and (e) taking blood lipid–regulating drugs.

### CCTA image acquisition

CCTA was performed on Siemens third-generation dual-source CT system (SOMATOM definition force; Siemens AG, Munich, Germany). Before scanning, the patient rested quietly for ≥ 15 min, and no patients took drugs such as β-blockers to slow down their heart rate. The scanning range was from the superior sternal fossa to the diaphragmatic surface of the heart. The prospective electrocardiogram gating sequence was used for scanning, and the scanning parameters were as follows: tube voltage, 100 kV; tube current, 228–300 mA; CT rotation time, 0.25 s; layer thickness, 0.75 mm; reconstruction interval, 0.50 mm; and display matrix, 512 × 512. Iodixanol injection (100 mL: 32 g; Qing Liming, NJCTTQ, China) was injected through the middle elbow vein. The injection volume was 60–80 mL, and the injection speed was 5–6 mL/s.

### Coronary artery reconstruction

The scanned images were transmitted to the Skviewer software program (Coronary System; Shukun Technology, Beijing, China). At first, each image was processed for image consistency to eliminate the impact of different window widths and window levels on the quality of the reconstructed image. Then, Coronary System was used for longitudinal and axial multiplanar reconstruction of the coronary artery. Finally, an experienced radiologist evaluated the image quality of coronary artery reconstruction, awarding a score of 1–4 points, respectively, for poor image quality (images were subsequently excluded), acceptable image quality, good image quality, and excellent image quality.

### Perivascular fat analysis

**Fig. 2:**
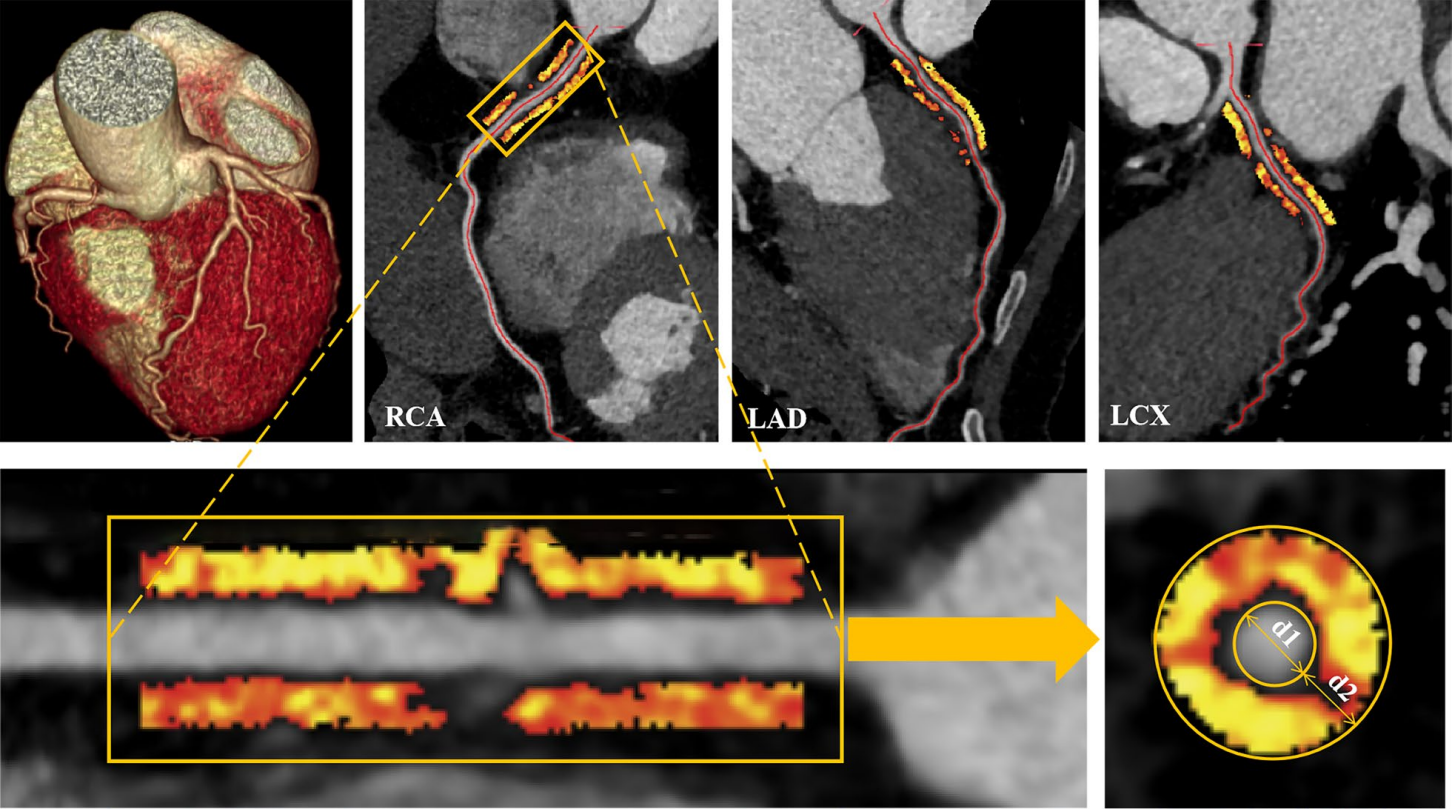
The PCAT was extracted by the Skviewer software automatically. *d1* diameter of the target vessel, *d2* extracted radial distance of PCAT

PCAT was extracted automatically by using the Skviewer software FAI intelligent analysis system (Skviewer FAI; Shukun Technology, Beijing, China) [12]. The volume and FAI of PCAT were measured using the method described by Oikonomou et al. [7]; the threshold value of PCAT ranged from − 190 to − 30 HU, the measured length was 40 mm, and the extracted radial distance was the average diameter of the target vessel. The segment analyzed was the proximal 40 mm of the left anterior descending (LAD) and left circumflex (LCX) arteries and the proximal 10–50 mm of the right coronary artery (RCA). The software could extract PACT volume and FAI automatically, and when the automatic extraction was inaccurate, we adjusted the extraction range manually. Subsequently, the PCAT volume and FAI were calculated by the software automatically (Fig. 2). The above measurement results were completed by an experienced radiologist independently. In order to test the consistency of the measurement results, 20% (32) of patients were randomly selected 1 month later, and a second measurement session was carried out by 2 radiologists. An intraobserver consistency test was carried out on the results measured by the same doctor, and an interobserver consistency test was carried out on the results measured by 2 doctors (Additional file 1).

### ICA and Gensini score calculation

According to the 2021 American College of Cardiology/American Heart Association angiography guidelines of the United States, the patients were punctured through the right femoral artery or radial artery with the conventional Seldinger standard method. The left and right coronary arteries were then successively imaged with multi-position irradiation imaging [13].

The ICA results were evaluated by lumen diameter stenosis (DS). The segment score was obtained by multiplying the corresponding scores of the vascular segment where the plaque was located by the score of stenosis. The sum of the total scores of each segment was the total Gensini score [14] (Table 1).

**Table 1:** Gensini scoring system

DS (%)	Score	Location	Corresponding score
0–25	1	LM	5.0
26–50	2	Proximal segment of LAD or LCX	2.5
51–75	4	Middle segment of LAD	1.5
76–90	8	Distal segment of LAD or LCX	1
91–99	16	RCA, PDA	1
100	100	D1, OM	1
		D2, PLV	0.5

*LM* left main, *LAD* left anterior descending, *LCX* left circumflex, *RCA* right coronary artery, *PDA* posterior descending artery, *OM* obtuse marginal, *D1* first diagonal, *D2* second diagonal, *PLV* posterior left ventricular branch

### LV function parameter acquisition

According to the current guidelines and diagnostic criteria set by the American Society of Echocardiography and European Association of Cardiovascular Imaging [15], echocardiographs were acquired by the Vivid E9 (GE Vingmed Ultrasound AS; Horten, Norway) echocardiography system with an M5S transducer (3.5 MHz). The patient adopted the left-lying position, laid still for 5 min, and breathed calmly; then, we imaged the long-axis section of the LV to obtain the interventricular septum (IVS), LV posterior wall (LVPW), LV end-diastole diameter (LVEDD), LV end-systolic diameter (LVESD), 1eft ventricular end-diastolic volume (LVEDV), and left ventricular end-systolic volume (LVESV), then calculated the LV fractional shortening (FS), LV ejection fraction (LVEF), LV mass (LVM), and LV mass index (LVMI). The calculation formulae used are as follows:

LVEF = (LVEDV − LVESV)/LVEDV × 100%

FS (%) = (LVEDD − LVESD)/LVEDD

LVM (g) = 0.8*1.04*[(IVS + LPWT + LVEDD)3 − LVESD3] + 0.6

LVMI (g/m^2^) = LVM/body surface area

Body surface area (m^2^) = 0.0061 × height (cm) + 0.0128 × weight (kg) − 0.1529

### Statistical analysis

All statistical analyses were performed using SPSS (SPSS Statistics for Windows Version 23.0; IBM Corporation, Armonk, NY, USA). The counting data were expressed in quantity and percentage, and the continuous variables were described using mean ± standard deviation (SD) or median (upper quartile, lower quartile) values. Two subgroup comparisons of continuous variables used 2 independent-samples Student’s *t*-tests or Mann–Whitney *U* tests. The correlation between PCAT volume or FAI and Gensini score and LV function parameters was assessed using Spearman’s correlation analysis. In order to eliminate the influence of interference factors, we used a partial correlation analysis to analyze the correlation between the PCAT volume or FAI and Gensini score and LV function parameters. *P* < 0.05 was considered to indicate statistical significance. The intragroup correlation coefficient (ICC) was used to evaluate intra- and interobserver consistency.

## Results

### Baseline characteristics

Initially, 197 participants with clinically suspected CAD were included. According to strict exclusion criteria, 159 patients (mean age, 64.55 ± 10.64 years; range, 43–89 years) were finally enrolled in this study (Fig. 1), including 104 men (mean age, 62.03 ± 10.58 years; range, 43–89 years) and 55 women (mean age, 69.33 ± 9.05 years; range, 51–85 years). Other baseline characteristics are shown in Table 2.

**Table 2:** Baseline characteristics of study participants

Characteristics	Result
Age (y)*	64.55 ± 10.64
*Gender*	
Male	104 (65.4%)
Female	55 (34.6%)
Height (cm)+	165 (160, 170)
Weight (kg)*	66.69 ± 11.21
BMI*	24.42 ± 3.12
Heart rate+	74 (66, 80)
Gensini score+	19 (10, 41)
*Risk factors of CAD*	
Hypertension	116 (73.0%)
SBP (mmHg)+	138 (126, 150)
DBP (mmHg)+	80 (72, 89)
Diabetes	63 (39.6%)
Glucose (mmol/L)+	5.39 (4.80, 6.76)
Dyslipidemia	95 (59.7%)
TC (mmol/L)+	4.12 (3.45–5.10)
TG (mmol/L)+	1.48 (1.11–2.23)
HDL-C (mmol/L)+	1.01 (0.88–1.23)
LDL-C (mmol/L)+	2.47 (1.88–3.06)
Smoking	58 (36.5%)
Drinking	24 (15.1%)
*Creatinine (mmol/L)+*	71 (62, 81)
TIMI = 3	
LAD	150 (94.3%)
LCX	157 (98.7%)
RCA	145 (91.2%)
*Echocardiography*	
IVS (mm)*	9.50 ± 1.13
LVPW (mm)*	9.42 ± 0.93
LVEDD (mm)*	49.82 ± 4.45
LVESD (mm)+	33.6 (31.5–35.9)
FS (%)+	31.4 (29.9–33.3)
LVEDV (mL)+	117.21 (101.62–132.00)
LVESV (mL)+	46.10 (39.40–54.10)
LVEF (%)+	58.81 (56.80–62.00)
LVM (g)+	167.11 (144.65–194.00)
LVMI (g/m2)+	99.76 (85.36–109.82)

*Data are shown as mean ± standard deviation values

+Data are presented as median values with the interquartile range in parentheses

*TIMI* thrombolysis in myocardial infarction (TIMI = 3 [complete perfusion] means that the coronary artery is fully developed and the blood flow is normal)

### Relationship between PCAT volume and CAD risk factors

**Fig. 3:**
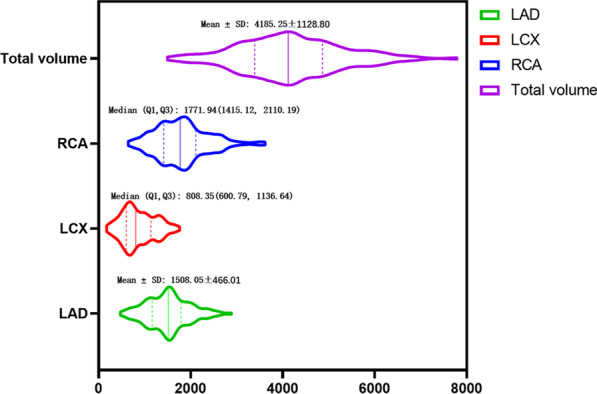
The distribution range of PCAT volume

The PCAT volumes of all participants were divided into 2 subgroups according to whether they had hypertension, diabetes, dyslipidemia, a smoking history, or a drinking history. Table 3 shows the distribution of volume among the subgroups. The results showed that there were statistically significant differences in the LCX volume and total volume between the subgroups (*P* = 0.028 and *P* = 0.025). The results of the other subgroups were not significant (*P* > 0.05). The distribution range of PCAT volume is shown in Fig. 3. [The PCAT intra- and interobserver consistencies were good (*P* < 0.001) (Additional file 1)].

**Table 3:** Relationship between PCAT volume and CAD-related risk factors

Group	n	LAD		LCX		RCA		Total volume	
		Volume	*P*	Volume	*P*	Volume	*P*	Volume	*P*
Total	159	1508.05 ± 466.01		808.35 (600.79, 1136.64)		1771.94 (1415.12, 2110.19)		4185.25 ± 1128.80	
Hypertension			0.678		0.954		0.175		0.599
Yes	116	1498.66 ± 429.52		871.45 ± 351.15		1843.89 ± 547.27		4214.00 ± 1045.14	
No	43	1533.38 ± 557.58		867.76 ± 390.23		1706.54 ± 609.47		4107.68 ± 1339.43	
Diabetes			0.705		0.304		0.563		0.840
Yes	63	1490.72 ± 391.45		1490.72 ± 391.45		1838.03 ± 523.76		4162.77 ± 910.17	
No	96	1519.43 ± 510.73		1519.43 ± 510.73		1786.21 ± 594.06		4200.01 ± 1256.18	
Dyslipidemia			0.654		0.499		0.071		0.506
Yes	95	1521.69 ± 444.34		854.50 ± 330.34		1858.07 ± 550.06		4234.26 ± 1073.34	
No	64	1487.81 ± 499.34		894.14 ± 403.53		1680.80(1335.61, 1915.44)		4112.50 ± 1211.47	
Smoking			0.327		0.415		0.150		0.145
Yes	58	1556.01 ± 497.75		776.65(635.17, 1235.13)		1892.12 ± 548.84		4357.67 ± 1120.09	
No	101	1480.51 ± 446.99		848.01 ± 360.07		1757.72 ± 572.72		4086.24 ± 1127.37	
Drinking			0.130		0.028		0.103		0.025
Yes	24	1640.77 ± 579.42		1037.84 ± 375.47		1980.53 ± 652.73		4659.15 ± 1202.05	
No	135	1484.46 ± 441.27		791.59 (585.98, 1093.43)		1775.85 ± 546.24		4101.00 ± 1098.65	

The data with normal distribution are described by mean ± standard deviation values and were tested with an independent-samples Student’s *t*-test, while those with non-normal distribution are described by median (upper quartile, lower quartile) values and were tested with the Mann–Whitney *U* test

### Relationship between FAI and CAD risk factors

**Fig. 4:**
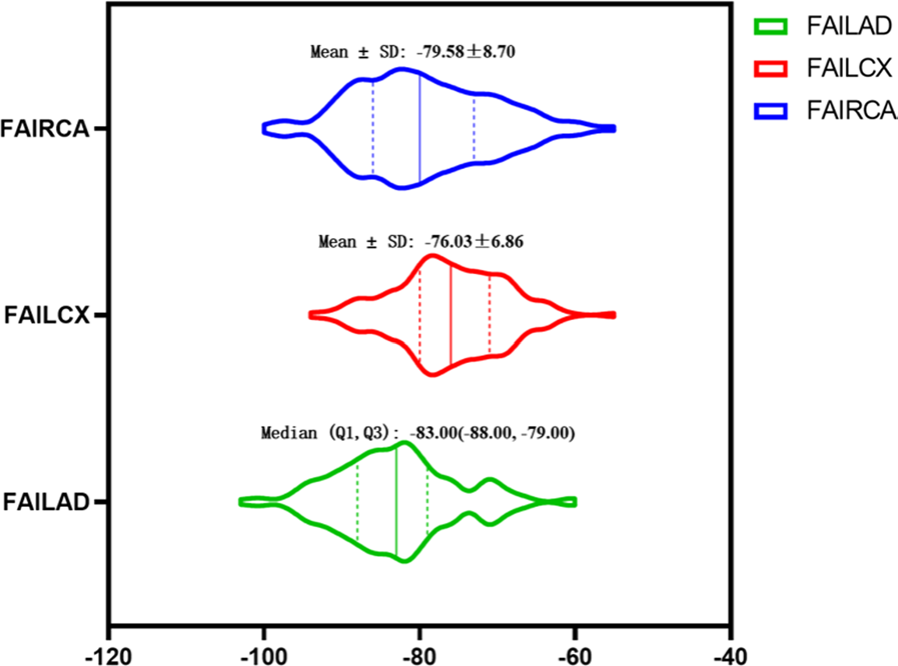
The distribution range of FAI

Table 4 shows the distribution of FAI among different subgroups. The results showed that there was no significant difference in FAI between the subgroups (*P* > 0.05). Figure 4 shows the distribution range of FAI.

**Table 4:** Relationship between FAI and CAD-related risk factors

Group	n	LAD		LCX		RCA	
		FAI	*P*	FAI	*P*	FAI	*P*
Total	159	− 83.00 (− 88.00, − 79.00)		− 76.03 ± 6.86		− 79.58 ± 8.70	
Hypertension			0.808		0.809		0.511
Yes	116	− 82.97 ± 7.49		− 76.11 ± 7.07		− 79.86 ± 8.56	
No	43	− 83.30 ± 8.45		− 75.81 ± 6.35		− 78.84 ± 9.11	
Diabetes			0.875		0.466		0.423
Yes	63	− 82.94 ± 6.95		− 75.54 ± 7.08		− 80.27 ± 7.72	
No	96	− 83.14 ± 8.25		− 76.35 ± 6.74		− 79.14 ± 9.30	
Dyslipidemia			0.474		0.073		0.584
Yes	95	− 82.69 ± 7.78		− 75.23 ± 6.60		− 79.27 ± 8.54	
No	64	− 83.59 ± 7.71		− 77.22 ± 7.13		− 80.05 ± 8.97	
Smoking			0.429		0.826		0.671
Yes	58	− 82.41 ± 8.08		− 76.19 ± 7.52		− 79.00 ± 8.42	
No	101	− 83.43 ± 7.55		− 75.94 ± 6.50		− 80.50(− 86.00, − 73.75)	
Drinking			0.746		0.670		0.880
Yes	24	− 82.58 ± 9.41		− 76.58 ± 8.02		− 79.83 ± 8.11	
No	135	− 83.14 ± 7.44		− 75.93 ± 6.67		− 79.54 ± 8.83	

The statistical parameters are consistent with those of Table 3

### Correlation analysis of PCAT volume or FAI and Gensini score or BMI

**Fig. 5:**
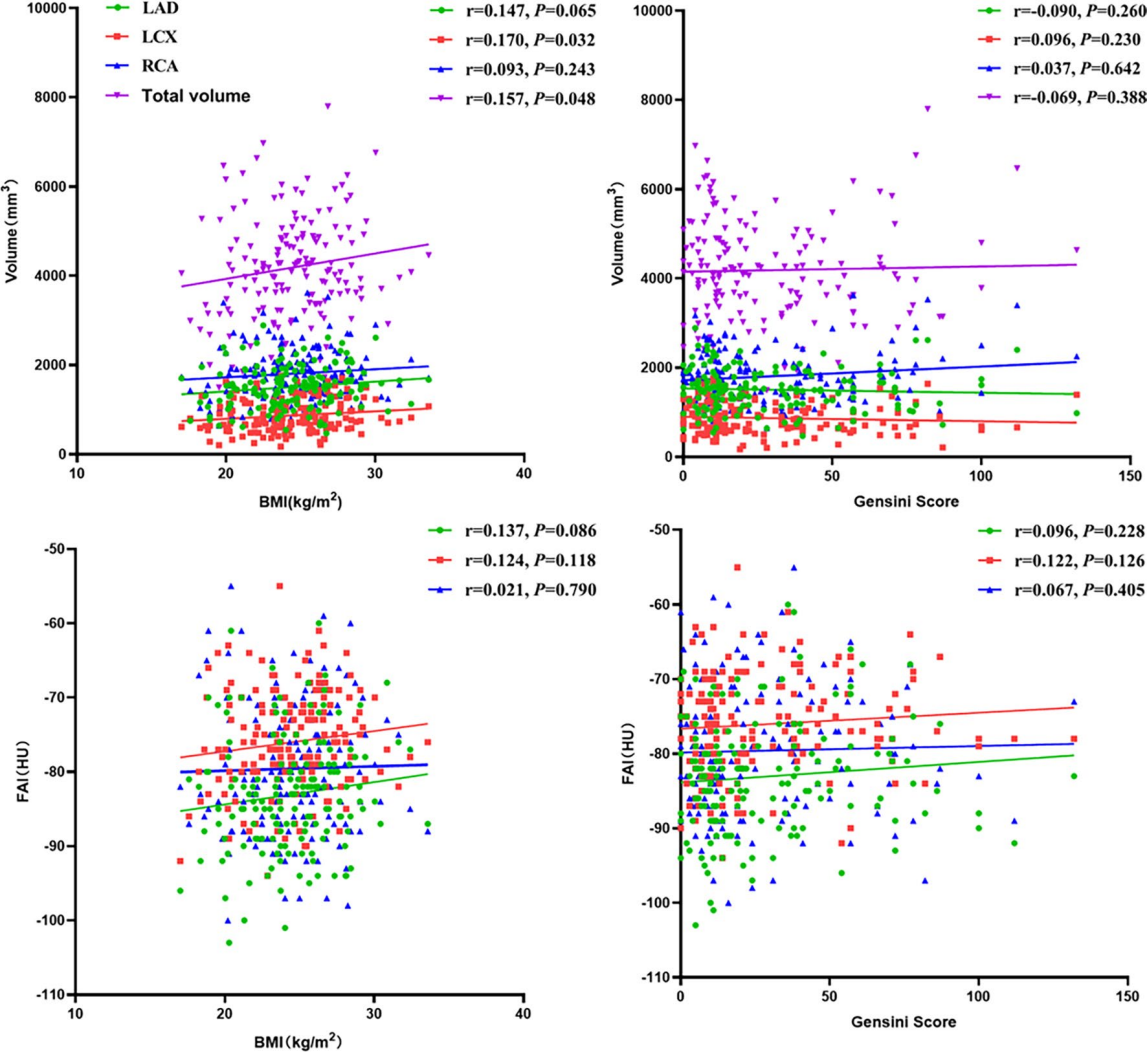
Correlation analysis of PCAT volume or FAI and Gensini score or BMI

Spearman’s correlation or Pearson’s analysis was used to analyze the correlation between PCAT and Gensini score and BMI. The results showed that the LCX and total volumes were positively correlated with BMI (r = 0.170, *P* = 0.032; r = 0.157, *P* = 0.048). However, there was no significant correlation between the LAD and RCA volumes and BMI and no correlation between FAI and BMI. The PCAT also was not correlated with the Gensini score, as shown in Fig. 5.

### Correlation analysis of PCAT volume or FAI and LV function parameters.

**Fig. 6:**
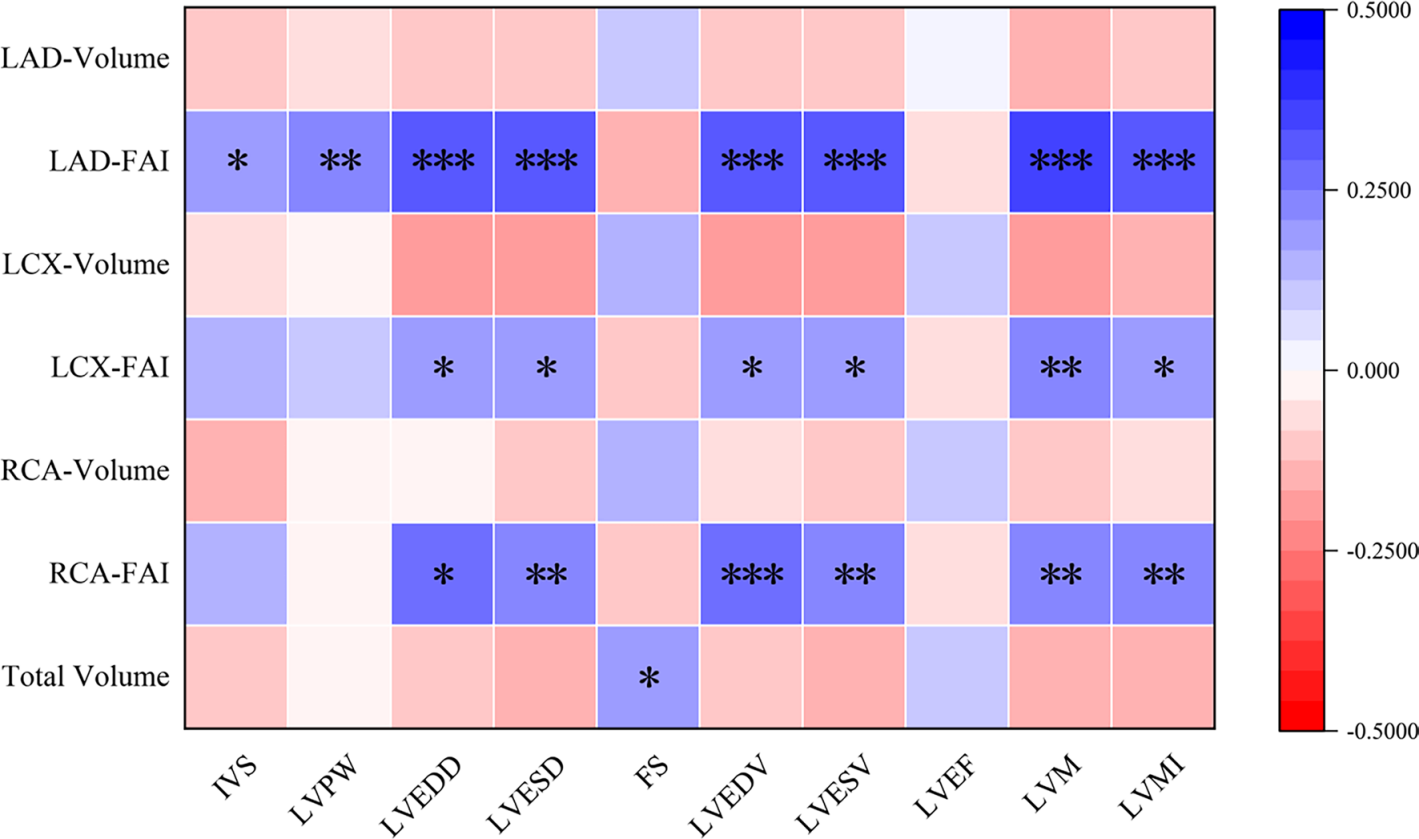
Partial correlation analysis results of PCAT and LV function parameters. The results are presented in the form of a matrix. The LAD-FAI was positively correlated with IVS (r = 0.203, *P* = 0.013), LVPW (r = 0.218, *P* = 0.008), LVEDD (r = 0.317, *P* < 0.001), LVESD (r = 0.298, *P* < 0.001), LVEDV (r = 0.317, *P* < 0.001), LVESV (r = 0.301, *P* < 0.001), LVM (r = 0.371, *P* < 0.001), and LVMI (r = 0.304, *P* < 0.001). The LCX-FAI was positively correlated with LVEDD (r = 0.199, *P* = 0.015), LVESD (r = 0.190, *P* = 0.021), LVEDV (r = 0.203, *P* = 0.013), LVESV (r = 0.197, *P* = 0.016), LVM (r = 0.220, *P* = 0.007), and LVMI (r = 0.172, *P* = 0.036). The RCA-FAI was positively correlated with LVEDD (r = 0.258, *P* = 0.002), LVESD (r = 0.238, *P* = 0.004), LVEDV (r = 0.266, *P* = 0.001), LVESV (r = 0.249, *P* = 0.002), LVM (r = 0.237, *P* = 0.004), and LVMI (r = 0.218, *P* = 0.008). The total volume was positively correlated with FS (r = 0.167, *P* = 0.042)

We performed a partial correlation analysis of control variables, excluding confounding factors such as age, gender, height, weight, BMI, smoking, drinking, heart rate, hypertension, diabetes and hyperlipidemia, then analyzed the correlation between PCAT and LV function parameters (Fig. 6).

## Discussion

While using CCTA to diagnose coronary artery stenosis, we can predict the prognosis of patients with CAD through the analysis of PCAT.

Some studies on the correlation between epicardial fat volume (EFV) and CAD reported that the increase of EFV was positively correlated with the risk of CAD [16, 17]. Severe CAD often leads to changes in cardiac function, and studies have shown that there is a correlation between EVF and LV function parameters [18, 19]. Thus, we analyzed the relationship between PCAT volume, Gensini score, and LV function parameters and found that there was no correlation between them. The possible reason for this is that the measurement methods are different. The extraction range of EFV is all adipose tissue from the aortic root to the apex of the heart, and the radial range of extraction of PCAT is equal to the diameter of the target vessel. Many factors are related to coronary heart disease, including obesity, diabetes, and hypertension [20, 21]. Previous studies have shown that EVF is correlated with traditional risk factors of coronary heart disease [22–24] and positively correlated with BMI [25]. However, some research results suggest that EVF can be an independent predictor of CAD [26] and is not associated with the traditional risk factors of CAD [27, 28]. Therefore, we analyzed the correlation between PCAT volume, BMI, and risk factors for CAD. Our results showed that PCAT volume did not correlate with BMI or risk factors for coronary heart disease. These differences in results may be related to different measurement methods, pathological changes in the natural course of the disease, lifestyle factors, and drug intervention [29, 30]. Our results showed that only the results of LCX and total volumes were statistically significant (*P* < 0.05). This may be related to the reduced amount of adipose tissue around the LCX; the method we adopted can cover a more complete vessel volume. The correlation shown with the total volume may be affected by LCX. In a study of pericoronary epicardial adipose tissue by Vos et al. [31], only the fat volume around LCX showed different results from LAD and RCA, which was similar to our results. Inflammation is a critical factor not only for the development but also the progression of atherosclerosis [1, 32, 33]. Inflammatory factors released by the arterial wall can induce lipolysis, inhibit lipogenesis, and promote perivascular edema [34]. Meanwhile, the cytokines secreted by adipose tissue play a significant role in atherogenesis and myocardial ischemia [35, 36]. These changes showed attenuation from lipids (close to − 190 Hu) to water (close to − 30 Hu) on CT [37, 38]. Therefore, coronary inflammation can be detected by FAI.

One study by Antonopoulos et al. [38] found that FAI was positively correlated with atherosclerotic plaque load, and a greater perivascular FAI was associated with higher inflammatory expression levels. Some other studies have found that FAI is related to coronary hemodynamic changes and myocardial ischemia; furthermore, FAI around culprit lesions was increased significantly in patients with acute myocardial infarction [39, 40]. Therefore, we analyzed the correlation between FAI and Gensini score and found that there was no correlation between PCAT FAI and Gensini score (*P* > 0.05). However, what triggered this result? It may be that we did not stratify the population according to the Gensini score, and there were only 26 patients (16%) with severe CAD (Gensini score > 60 points) in our study population. Meanwhile, some studies have shown that FAI can present dynamic changes with the prognosis of the disease, and statins, aspirin, or anti-diabetic drugs can also alter the FAI [41, 42]. In addition, coronary artery calcification is closely correlated with atherosclerotic plaque formation and cardiovascular disease, but some studies have shown the potential impact of calcification on machine learning [43, 44]. These may cause our results to be different from others.

Further analysis found that there was no significant difference between FAI and BMI (LAD: r = 0.137, *P* = 0.086; LCX: r = 0.124, *P* = 0.118; RCA: r = 0.021, *P* = 0.790). Between the results of FAI and risk factors of CAD, there was no relationship. These findings can further support the idea that FAI is a reliable imaging index to quantify coronary artery inflammation and is not affected by other risk factors [45]. The study of Antonopoulos et al. [38] found that FAI was not associated with traditional cardiovascular risk factors, similar to our results.

Since the parameters of LV function are affected by individual differences [46, 47], we analyzed the PCAT and LV function parameters by partial correlation analysis to exclude the effects of interference factors. There was no correlation between PCAT and LV function parameters, but a significant correlation between FAI and LV function parameters existed. This may be because the coronary artery and myocardium have no obvious boundary, and the PCAT can release cytokines that reach the myocardium and coronary artery through paracrine signaling mechanisms, which results in changes in myocardial and LV function [48–50]. Hoshino et al. [40] mentioned that FAI was associated with cardiac mass, and some studies have reported a correlation between LV function and EAT [51, 52]; notably, these results are similar to ours. In addition, studies have shown that LV dysfunction and increased FAI (> 70.1 HU) can significantly increase the cardiac mortality [42, 53]. Our results suggest that cardiac mortality caused by increased FAI may be due to changes in LV function.

There are several limitations to this study that should be pointed out. First, this study is a single-center retrospective analysis with a small sample size that failed to obtain the treatment and lifestyle intervention details of patients. Second, in our study, the number of patients with severe CAD was small, and we did not stratify the severity of patients, which may lead to bias in our results. Third, previous studies have shown that CAC is a reliable and independent predictor of future cardiovascular events [54], so we will evaluate the relationship between calcification and PCAT in future research. Fourth, some studies have shown that there are differences in FAI between different genders [55], and women account for less than half of the population in our study (34.6%), which may also cause deviation in the results. Further research is needed to identify sex-specific variables that explain the correlation between PCAT and LV function accurately.

## Conclusion

The results showed that there was no correlation between PCAT volume, FAI, and the severity of CAD, but there was a positive correlation between FAI and LV function parameters, and the correlation involving LAD was the strongest. Moreover, our study found that FAI has no significant relationship with BMI or traditional risk factors of CAD. It further supports that FAI can be used as a reliable imaging index of coronary artery inflammation, potentially playing a significant role in clinical diagnosis and evaluation of CAD.

## Supplementary Information

**Additional file 1.** The PCAT intra- and interobserver consistencies.

## Data Availability

The datasets generated and/or analyzed during the current study are not publicly available to protect patient privacy but are available from the corresponding author upon reasonable request.

## Abbreviations

CAD: Coronary artery disease
FAI: Pericoronary fat attenuation index
CCTA: Coronary computed tomography angiography
PCAT: Pericoronary adipose tissue
ICA: Invasive coronary angiography
BMI: Body mass index
LAD: Left anterior descending
LCX: Left circumflex
RCA: Right coronary artery
DS: Diameter stenosis
IVS: Inter ventricular septum
LVPW: Left ventricular posterior wall
LVEDD: Left ventricular end-diastole diameter
LVESD: Left ventricular end-systolic diameter
LVEDV: Left ventricular end-diastolic volume
LVESV: Left ventricular end-systolic volume
FS: Fractional shortening
LVEF: Left ventricular ejection fraction
LVM: Left ventricular mass
LVMI: Left ventricular mass index
EFV: Epicardial fat volume
