# Supplementary material for: The correlation of pericoronary adipose tissue with coronary artery disease and left ventricular function
Source: BMC Cardiovasc Disord. 2022 Sep 6;22:398. doi: 10.1186/s12872-022-02843-y (PMC9446702; doi:10.1186/s12872-022-02843-y)
Supplement: Supplementary file 1 — Additional file 1. The PCAT intra- and interobserver consistencies. [file 12872_2022_2843_MOESM1_ESM.docx]

Additional file 1. The PCAT intra- and interobserver consistencies.

| PCAT | intraobserver consistency | |  | interobserver consistency | |
| --- | --- | --- | --- | --- | --- |
|  | ICC | P |  | ICC | P |
| LAD-volume | 0.982 | ＜0.001 |  | 0.991 | ＜0.001 |
| LAD-FAI | 0.954 | ＜0.001 |  | 0.996 | ＜0.001 |
| LCX-volume | 0.991 | ＜0.001 |  | 0.986 | ＜0.001 |
| LCX-FAI | 0.995 | ＜0.001 |  | 0.977 | ＜0.001 |
| RCA-volume | 0.986 | ＜0.001 |  | 0.972 | ＜0.001 |
| RCA-FAI | 0.996 | ＜0.001 |  | 0.982 | ＜0.001 |
| Total-volume | 0.992 | ＜0.001 |  | 0.976 | ＜0.001 |

ICC=intragroup correlation coefficient
